# Radiographic Assessment of Normal Periapical Bone Density: A Descriptive Analysis Based on Digital Imaging

**DOI:** 10.1155/ijod/8200566

**Published:** 2026-07-26

**Authors:** Motahare Baghestani, Amirhossein Zirehpour, Mostafa Azimzadeh, Maryam Kazemipoor

**Affiliations:** ^1^ Department of Dentomaxillofacial Radiology, School of Dentistry, Shahid Sadoughi University of Medical Sciences, Yazd, Iran, ssu.ac.ir; ^2^ Department of Endodontics, School of Dentistry, Shahid Sadoughi University of Medical Sciences, Yazd, Iran, ssu.ac.ir

## Abstract

**Introduction:**

Accurate evaluation of alveolar bone density is essential for dental diagnosis and treatment planning. Panoramic radiographs offer relative estimates of bone density. Due to limited data in Iranian populations, this study assessed the radiographic density of normal periapical bone using panoramic imaging.

**Materials and Methods:**

This descriptive cross‐sectional study analyzed digital panoramic radiographs at three anatomical regions—anterior, premolar, and molar—in both jaws. A standardized 1.5 mm × 1.5 mm region of interest (ROI) was positioned within 2 mm of the apical third of each tooth. Bone density was quantified as gray values (arbitrary units [AU]) using Digora software. Demographic data were collected, and statistical comparisons between groups were performed using independent *t*‐tests and one‐way ANOVA at a significance level of *α* = 0.05.

**Results:**

In 458 panoramic radiographs (212 males, 246 females; ages 20–50), bone density was significantly higher in males, older individuals (35–50), and the mandible compared to the maxilla. Density decreased from anterior to molar regions. The anterior mandible showed the highest values, with notable differences from the anterior maxilla, while premolar and molar regions showed no significant interjaw differences.

**Conclusion:**

Panoramic radiographs revealed consistent patterns in periapical bone density. Density was higher in males, older adults, and the mandible compared to the maxilla. The anterior mandible showed the highest values overall. In the maxilla, bone density increased from anterior to posterior regions. These findings support baseline assessment and suggest further validation with 3D imaging.

## 1. Introduction

Bone is a highly active biological tissue that continuously adapts to physiological and mechanical demands through the coordinated processes of formation and resorption. These ongoing remodeling activities are essential for preserving skeletal strength, structural stability, and mineral homeostasis. Disturbances in the equilibrium between bone formation and bone breakdown may result in changes in bone mineral density (BMD), a parameter that reflects the amount of mineralized bone present within a given volume of bone tissue [1]. Systemic conditions such as postmenopausal osteoporosis, osteoarthritis, fracture healing, and implant‐related bone remodeling significantly influence BMD [2]. Additionally, endocrine and hematologic disorders—including hyperparathyroidism, hyperthyroidism, hypothyroidism, Cushing’s syndrome, thalassemia, rickets, and sickle cell anemia—can cause marked changes in bone density [3]. The alveolar bone, a specialized part of the craniofacial skeleton, plays a critical role in supporting teeth and maintaining oral function. It exists in both the maxilla and mandible, with the maxillary alveolar bone generally being thinner and more porous than its mandibular counterpart [4]. A gradual decline in BMD is a natural consequence of aging, further exacerbated by risk factors such as hormonal deficiencies, reduced physical activity, inadequate calcium and vitamin D intake, inflammatory diseases, corticosteroid use, smoking, and alcohol consumption [5]. Conversely, lifestyle modifications such as regular physical exercise and plant‐based or balanced diets have been shown to improve bone mineralization, particularly in middle‐aged and postmenopausal populations [6]. The characterization of alveolar bone density provides valuable information for a wide range of dental applications. It supports clinical decision‐making by helping practitioners evaluate treatment feasibility, optimize therapeutic planning, and monitor outcomes following interventions including implant placement, periodontal procedures, and orthodontic tooth movement [7]. Moreover, radiographic evidence of reduced jawbone density may serve as an early indicator of systemic bone loss, such as osteoporosis, thereby aiding in timely referral and management [8]. In clinical practice, bone density measurements are also valuable in evaluating temporomandibular joint disorders, periapical lesion differentiation, and craniofacial manifestations of systemic diseases [9]. While conventional radiographic films were historically the primary imaging modality, advancements in digital imaging and densitometric technologies have enhanced the precision and accessibility of bone density evaluation [10]. Cone‐beam computed tomography (CBCT) offers high‐resolution three‐dimensional imaging of hard tissues, including the alveolar bone, and is increasingly used in dental diagnostics [11]. However, due to cost, accessibility, and radiation concerns, panoramic radiography remains a widely used, noninvasive, and cost‐effective tool in routine dental assessments [7]. Despite their relatively limited spatial resolution, panoramic radiographs remain valuable for the assessment of skeletal characteristics within the maxillofacial region. Quantitative and qualitative indices obtained from these images, including measurements of mandibular cortical dimensions and morphology, have been proposed as screening tools for identifying individuals at risk of decreased BMD and osteoporosis‐related fractures, particularly among postmenopausal women [7]. Given the limited data on normative alveolar bone density in Iranian populations, this study was designed as a baseline investigation to evaluate the radiographic density of the normal periapical bone across different jaw regions. By considering demographic and anatomical variables, this research aims to contribute reference data that may enhance diagnostic accuracy and inform clinical decision‐making in dental and systemic health contexts.

## 2. Materials and Methods

This cross‐sectional study aimed to determine the normal radiographic bone density in adult patients using digital panoramic radiographs. A total of 458 images were randomly selected from the 2023–2024 archive of the Radiology Department, Faculty of Dentistry, Shahid Sadoughi University of Medical Sciences, Yazd. Sampling was performed using simple random sampling without replacement. To ensure independence, only one radiograph per patient was included; in cases with multiple eligible images, the earliest qualifying radiograph within the study period was selected. To ensure adequate statistical power, a minimum of 455 radiographs were targeted based on prior standard deviation estimates, and a total of 458 images were analyzed to accommodate potential exclusions. Inclusion criteria required that radiographs display periapical regions free of inflammatory, neoplastic, or developmental osseous lesions and be free of technical errors (e.g., positioning or exposure problems) or anatomical superimpositions (e.g., oropharyngeal airway over maxillary apices) that could interfere with accurate measurements. Exclusion criteria encompassed patients with systemic diseases known to affect jawbone density, including endocrine disorders (e.g., hyperparathyroidism, hypoparathyroidism, hyperthyroidism, Cushing’s syndrome) and metabolic and genetic bone diseases (e.g., osteoporosis, osteopetrosis, rickets, hypophosphatasia, hypophosphatemia, renal osteodystrophy, sickle cell anemia, thalassemia, and osteomalacia).

Panoramic radiographs of patients aged 20–50 years were acquired using a Planmeca Ec Proline system (Helsinki, Finland) with exposure parameters of 18 s, 6 mA, and 66 kVp. Images were exported and analyzed using Digora software to evaluate bone density, expressed as mean gray values in arbitrary units (AU). The region of interest (ROI) was defined as a standardized 1.5 mm × 1.5 mm square located within 2 mm of the apical third of the root. This location was selected because the periapical trabecular bone adjacent to the root apex is clinically relevant, relatively homogeneous, and less likely to be influenced by anatomical landmarks or cortical bone structures that could affect density measurements. To minimize anatomical variability, ROIs were placed only in areas free from radiographic pathology, root overlap, and adjacent anatomical structures. Special attention was given to the anterior mandibular region to avoid the radiopaque projection of the cervical vertebrae. Radiographs showing cervical vertebral superimposition over the intended ROI were excluded from analysis, and ROI placement was adjusted to include only homogeneous trabecular bone free from projection artifacts.

Measurements were obtained from the anterior (central, lateral, and canine teeth), premolar (first and second premolars), and molar (first and second molars) regions of both jaws using a standardized ROI placement protocol applied to all images. Demographic variables including age, sex, jaw, anatomical region, and bone density were recorded using structured checklists.

To assess measurement reproducibility, 50 radiographs (~11% of the sample) were randomly selected for intraobserver reliability testing. The same examiner repeated the full measurement protocol after a washout period of at least 2 weeks. Images were rerandomized and blinded to prior measurements, and identical ROI placement criteria and software settings were used during both assessment sessions to ensure methodological consistency and reduce measurement variability.

### 2.1. Statistical Analyses

After bone density measurements, the data were analyzed using SPSS Version 20. Descriptive statistics were generated, and comparisons were performed using ANOVA and independent *t*‐tests. Statistical significance was set at *p* < 0.05.

### 2.2. Missing Data

All variables required for the analysis (age, sex, jaw location, dental region, and bone density measurements) were available for all included radiographs. Therefore, no missing data were identified, and no imputation procedures were required.

## 3. Results

A total of 458 panoramic radiographs met the eligibility criteria and were included in the final analysis. Radiographs presenting inflammatory, developmental, or neoplastic osseous lesions, technical errors, anatomical superimpositions interfering with measurements, or a documented history of systemic diseases affecting bone density were excluded during the screening process. All included radiographs contained complete demographic and radiographic information and were available for analysis. Because data were collected retrospectively from an image archive and all eligible cases were analyzed, no loss to follow‐up occurred.

In this study, a total of 458 panoramic radiographic datasets were analyzed, including 246 images from female patients and 212 from male patients. The sample comprised 200 cases from individuals aged 20–34 years and 258 cases from those aged 35–50 years. Anatomically, 256 radiographs were obtained from the mandible and 202 from the maxilla. Based on tooth type, 152 radiographs corresponded to the anterior teeth, 153 to the premolars, and 153 to the molars. Specifically, the mandibular region included 86 anterior, 86 premolar, and 84 molar radiographs, while the maxillary region comprised 66 anterior, 67 premolar, and 69 molar radiographs. Bone density was evaluated using Digora software, with measurements expressed as mean gray values in AU. As shown in Table 1, the mean bone density was significantly higher in male patients (74.66 AU) compared to female patients (66.24 AU).

**Table 1:** Mean bone density by gender.

Variable	Mean (AU)	Standard deviation (SD)	Minimum (AU)	Maximum (AU)	*p*‐Value ^∗^
Women	66.24	18.59	30	122	0.0001
Men	74.66	17.91	30	130	

^∗^Independent *t*‐tests.

Among the analyzed samples, 200 cases belonged to the age group of 20–34 years and 258 cases belonged to the age group of 35–50 years. The analysis revealed that the mean bone density in the 20–34‐year age group (67.82 AU) was significantly lower compared with that in the 35–50‐year age group (71.94 AU; Table 2).

**Table 2:** Mean bone density by age group.

Variable	Mean (AU)	Standard deviation (SD)	Minimum (AU)	Maximum (AU)	*p*‐Value ^∗^
20–34	67.82	19.58	30	130	0.019
35–50	71.94	17.89	30	122	

^∗^Independent *t*‐tests.

Among the analyzed samples, 256 radiographs were obtained from the mandible and 202 radiographs from the maxilla. The analysis demonstrated that the mean bone density in the maxilla (65.04 AU) was significantly lower compared with that in the mandible (74.16 AU; Table 3).

**Table 3:** Mean bone density by jaw type.

Variable	Mean (AU)	Standard deviation (SD)	Minimum (AU)	Maximum (AU)	*p*‐Value ^∗^
Mandible	74.16	18.96	35	130	0.0001
Maxilla	65.04	17.19	30	115	

^∗^Independent *t*‐tests.

Regardless of the jaw type (maxilla or mandible), 152 radiographs corresponded to the anterior region, 153 to the premolar region, and 153 to the molar region. The analysis revealed that the mean bone density was highest in the anterior region, whereas in the molar region, it was significantly lower compared with the other regions (Table 4).

**Table 4:** Mean bone density by dental region.

Variable	Mean (AU)	Standard deviation (SD)	Minimum (AU)	Maximum (AU)	*p*‐Value ^∗^
Anterior	73.46	18.76	40	130	0.019
Premolar	69.42	17.11	35	115	
Molar	67.56	16.78	30	110	

^∗^ANOVA.

In the analyzed samples, the mean bone density of both jaws was compared across three regions: anterior (86 mandibular and 66 maxillary), premolar (86 mandibular and 67 maxillary), and molar (84 mandibular and 69 maxillary). As shown in Table 5, a statistically significant difference in mean bone density was observed only in the anterior region, where the mandibular anterior region demonstrated a higher mean density compared with the maxillary anterior region. No significant differences were detected in the premolar and molar regions.

**Table 5:** Mean bone density in different jaw regions.

Variable	Mean (AU)	Standard deviation (SD)	Minimum (AU)	Maximum (AU)	*p*‐Value ^∗^
Mandibular anterior	82.41	20.70	40	130	0.0001
Maxillary anterior	61.80	14.02	40	88	
Mandibular premolar	71.67	17.85	35	114	0.075
Maxillary premolar	66.53	18.15	35	115	
Mandibular molar	68.27	15.06	45	110	0.586
Maxillary molar	66.71	18.74	30	100	

^∗^Independent *t*‐tests.

Among the analyzed samples (Table 6), the mean bone density of the mandibular anterior region was significantly different compared with other jaw regions. The mandibular premolar region showed a significant difference in bone density only with the maxillary anterior region, and the mandibular molar region also demonstrated a significant difference compared with the maxillary anterior region.

**Table 6:** Correlation of mean bone density among different jaw regions.

Jaw region	Jaw region	*p*‐Value ^∗^
Mandibular anterior	Mandibular premolar	0.0001
	Mandibular molar	0.0001
	Maxillary anterior	0.0001
	Maxillary premolar	0.0001
	Maxillary molar	0.0001
Mandibular premolar	Mandibular molar	0.21
	Maxillary anterior	0.001
	Maxillary premolar	0.75
	Maxillary molar	0.82
Mandibular molar	Maxillary anterior	0.02
	Maxillary premolar	0.54
	Maxillary molar	0.58
Maxillary anterior	Maxillary premolar	0.12
	Maxillary molar	0.1
Maxillary premolar	Maxillary molar	0.95

^∗^ANOVA.

## 4. Discussion

In the present study, radiographic bone density was evaluated in 458 panoramic images, and the results showed that men exhibited significantly higher bone density than that of women. This observation is consistent with previous investigations: Mansuy et al. [12] reported lower bone density in women compared with men, and Di Stefano et al. [13] similarly demonstrated significant sex‐related differences in trabecular bone density. The reduced density in women is generally attributed to hormonal influences and the greater skeletal mass typically observed in men [2].

Interestingly, our analysis showed higher periapical bone density values among individuals aged 35–50 years compared with those aged 20–34 years. This observation should be interpreted with caution as it contrasts with the well‐established pattern of age‐related BMD decline reported in the broader literature. For example, Chugh et al. [14] found no significant association between age and bone density in adults aged 20–40 years, whereas Wakimoto et al. [15] observed age‐related reductions primarily among women in older age groups, particularly after menopause. The apparent discrepancy may be explained by the relatively narrow age range of our study population (20–50 years), which largely excludes the age groups in which substantial age‐related bone loss is most commonly observed. Furthermore, the cross‐sectional design does not permit the evaluation of longitudinal changes in bone density, and the findings may have been influenced by selection bias or unmeasured confounding factors such as systemic health status, nutritional factors, physical activity, and other lifestyle characteristics. Therefore, the observed association should not be interpreted as evidence of increasing bone density with age but rather as a population‐specific finding that warrants confirmation in larger studies with broader age distributions and longitudinal follow‐up.

The present findings showed that the periapical bone density was greater in the mandible than in the maxilla. This observation is consistent with previous investigations that have reported denser osseous structures in the mandibular arch [14–16]. The difference may be attributed to the structural and functional characteristics of the two jaws [16]. The mandible is subjected to substantial masticatory loading and contains a thicker cortical bone, whereas the maxilla exhibits a more trabecular architecture and distributes forces through the surrounding craniofacial skeleton [15, 16]. These anatomical distinctions may contribute to the density differences observed in the present study.

In our cohort, mandibular periapical bone density was significantly greater than maxillary density, in agreement with prior reports by Park et al. [16] and Chugh et al. [14]. Beyond these studies, multiple CT/CBCT investigations and reviews consistently rank regional jaw densities with the anterior mandible as the highest and the posterior maxilla as the lowest, reinforcing the mandible–maxilla gradient observed here [17–19]. Biomechanically, the mandible, an independent U‐shaped bone with substantial muscular attachments, undergoes greater bending and torsional stresses during mastication, favoring thicker cortical plates and coarser trabeculae; the maxilla, integrated into the midface, distributes occlusal forces through craniofacial buttresses and thus presents a thinner cortex and a more trabecular architecture [20, 21]. CBCT morphometric work further documents thinner cortical plates at many maxillary sites compared with mandibular sites, aligning the anatomy with the observed density gradient [22].

A regional pattern was identified, with the highest density values occurring in the anterior areas and lower values observed in the premolar and molar regions. Differences in local anatomy, trabecular organization, and functional loading may explain these findings [20–23]. The ROI was deliberately positioned within a standardized 1.5 mm× 1.5 mm area adjacent to the apical third of the root because this location is clinically relevant for assessing the periapical bone and is less affected by variations in cortical bone thickness. Consequently, the measurements primarily reflected trabecular bone characteristics rather than cortical bone morphology. While the use of a standardized ROI protocol enhanced measurement consistency and reproducibility across all radiographs, regional anatomical variability—including differences in trabecular architecture, proximity to anatomical landmarks, root morphology, and surrounding osseous structures—may still have influenced the recorded density values. An additional consideration when interpreting panoramic gray‐value measurements is the potential influence of superimposed anatomical structures. In particular, the radiopaque projection of the cervical vertebrae may overlap the anterior mandibular (infracanine) region and artificially increase the apparent radiopacity of the underlying trabecular bone. To minimize this source of measurement bias, all radiographs were evaluated before analysis, and images demonstrating cervical vertebral superimposition or other anatomical overlaps affecting the planned ROI were excluded. In addition, ROIs were placed only within visually homogeneous trabecular bone adjacent to the root apex, following a standardized protocol. Nevertheless, because panoramic radiography is a two‐dimensional projection technique, the complete elimination of projection‐related artifacts cannot be guaranteed. This limitation should be considered when interpreting regional differences in bone density and further supports the use of three‐dimensional imaging modalities, such as CBCT, in future validation studies. Comparable regional gradients have been reported in panoramic, CT, and CBCT studies, which generally identify the anterior mandible as one of the densest regions and the posterior maxilla as among the least dense [17, 24].

Regionally, mandibular bone density peaked in the anterior segment and declined toward the premolar and molar areas, whereas maxillary density was lowest anteriorly and increased posteriorly. These gradients differ from those reported by Chugh et al. [14] and Park et al. [16], likely due to methodological and anatomical differences. In particular, our ROI sampled a small periapical trabecular region, whereas previous investigations frequently evaluated inter‐radicular areas or included a greater contribution from cortical bone. Furthermore, panoramic gray‐value measurements are not directly comparable with CT‐ or CBCT‐derived density metrics [25]. Local anatomical factors may also affect small apical ROIs; for example, the anterior maxilla is characterized by a thin labial cortical plate and proximity to the nasopalatine canal and nasal floor, whereas posterior maxillary regions may be influenced by the sinus wall and floor [26, 27]. These considerations highlight the importance of standardized ROI selection when interpreting radiographic density measurements and underscore the potential influence of anatomical variability on regional bone density assessments.

Between the jaws, a significant density difference was limited to the anterior region: mandibular anterior sites exceeded maxillary anterior sites, whereas premolar and molar regions did not differ significantly. In contrast, Fuh et al. [28] reported no anterior mandibular–maxillary difference using three‐dimensional CBCT imaging. Discrepancies likely reflect methodology: our panoramic gray‐value measures from small apical‐third ROIs mainly sample trabecular bone, whereas CBCT often analyzes larger mixed cortical–trabecular volumes [24]. Also, cross‐modality comparability is limited because CBCT “HU” are device/protocol‐dependent while panoramic gray values are nonstandardized. Superimposition was mitigated via prespecified exclusions, but these can reduce sample size and bias comparisons—so report exclusion counts per site [25] and interpret no significant contrasts with effect sizes, 95% CIs, and stated multiple‐comparison adjustments.

Interindividual variation in bone density likely reflects a combination of metabolic, lifestyle, and racial/ethnic factors. Lifestyle exposures, including dietary calcium intake, habitual physical activity, and smoking, are associated with measurable differences in BMD, a pattern supported by recent meta‐analyses showing modest gains with resistance/combined exercise and dose‐dependent BMD deficits in smokers; by contrast, increases from calcium intake are generally small and nonprogressive [29, 30]. Additionally, large cohorts and review data consistently report race/ethnicity‐related differences in BMD after adjusting for body size and composition [31].

The increasing integration of digital technologies and artificial intelligence (AI) into dental imaging is expected to further enhance the accuracy and efficiency of radiographic assessment. Recent studies have demonstrated that AI‐based systems can achieve high diagnostic performance in the detection and classification of dentomaxillofacial findings on radiographic images, supporting clinicians in image interpretation and decision‐making. In this context, establishing reliable reference values for normal periapical bone density, as presented in the current study, may provide valuable baseline data for the development, training, and validation of future AI‐assisted diagnostic models. As digital dentistry continues to evolve, the combination of quantitative radiographic analysis and machine learning approaches may contribute to a more objective, standardized, and reproducible assessment of periapical bone health [32, 33].

Beyond their diagnostic value in evaluating the normal alveolar bone, baseline radiographic density measurements may have important applications in implant dentistry. Recent studies have suggested that longitudinal changes in radiographic bone density around dental implants can provide useful information regarding peri‐implant bone remodeling and the progression of osseointegration during healing [34, 35]. In this context, establishing normative reference values for periapical bone density in healthy individuals may offer a useful baseline against which postoperative radiographic changes can be interpreted. Although panoramic gray values cannot directly quantify osseointegration and are not equivalent to quantitative CT‐derived bone density measurements, they may assist clinicians in identifying expected patterns of bone adaptation or detecting deviations that warrant further investigation. Future prospective studies integrating baseline radiographic density assessments with serial postoperative imaging and clinical implant outcomes are needed to determine the value of this approach for monitoring peri‐implant healing and long‐term implant success.

Several limitations should be considered when interpreting the findings of this study. First, the cross‐sectional design precludes the assessment of temporal or causal relationships between demographic factors and bone density. Second, bone density was estimated using gray values obtained from panoramic radiographs rather than quantitative computed tomography, dual‐energy X‐ray absorptiometry, or CBCT, which may limit measurement precision and cross‐modality comparability. In addition, panoramic radiographic gray values are not standardized across imaging systems and are influenced by factors such as image acquisition parameters, magnification, superimposition of anatomical structures, and image processing algorithms. As a two‐dimensional imaging modality, panoramic radiography also lacks the volumetric information provided by CBCT, which may offer a more accurate assessment of bone characteristics. Therefore, future studies employing three‐dimensional imaging modalities, particularly CBCT, are warranted to validate and extend the present findings. Third, information regarding lifestyle factors known to influence bone density, including smoking status, alcohol consumption, dietary calcium intake, physical activity, and body mass index, was not available in the radiographic archive and therefore could not be controlled as potential confounders. Finally, the study population was derived from a single academic dental center in Iran, which may limit the generalizability of the findings to other populations.

## 5. Conclusion

This study established reference values for normal periapical bone density in an Iranian population using digital panoramic radiography. Significant differences were observed according to sex, age group, jaw location, and dental region. Higher density values were generally associated with male sex, older age, and mandibular sites, particularly in the anterior region. These findings suggest that demographic and anatomical factors should be considered when interpreting radiographic bone density measurements. Although panoramic radiography provides a practical and widely available method for evaluating bone characteristics, further studies using three‐dimensional imaging techniques are warranted to confirm and expand upon these observations.

**Nomenclature**
BMD: Bone mineral density
CBCT: Cone beam computed tomography
CI: Confidence interval
CT: Computed tomography
HU: Hounsfield unit
ROI: Region of interest.

## Author Contributions

**Maryam Kazemipoor:** conceptualization, methodology, formal analysis, writing – original draft, writing – review and editing, project administration. **Mostafa Azimzadeh**: conceptualization, methodology, formal analysis, writing – original draft, writing – review and editing. **Amirhossein Zirehpour**: investigation, data curation. **Motahare Baghestani**: formal analysis, writing – review and editing. All authors have contributed to the interpretation of the data.

## Funding

The present study was supported by the Shahid Sadoughi University of Medical Sciences (Grant 1304).

## Disclosure

All authors have read, critically reviewed the manuscript, approved the final version for publication, and agreed to be accountable for all aspects of the work. Maryam Kazemipoor (corresponding author) had full access to all of the data in this study and takes complete responsibility for the integrity of the data and the accuracy of the data analysis. The funders had no role in the design of the study; in the collection, analyses, or interpretation of data; in the writing of the manuscript; or in the decision to publish the results. All AI‐assisted edits were carefully reviewed, verified, and revised by the authors to ensure accuracy, clarity, and alignment with the original scientific content.

## Ethics Statement

This study has been approved by Ethic Committee Research Shahid Sadoughi University of Medical Sciences, Yazd (IR.SSU.DENTISTRY.REC.1403‐1402.054) and has therefore been performed in accordance with the ethical standards laid down in the 1964 Declaration of Helsinki and its later amendments. Patient anonymity and confidentiality were strictly maintained. All panoramic radiographs were obtained from the institutional archive and were analyzed anonymously. No personally identifiable information was collected, recorded, or reported.

## Consent

The authors have nothing to report.

## Conflicts of Interest

The authors declare no conflicts of interest.

## Data Availability

The data supporting the findings of this study are available from the corresponding author upon reasonable request. The data are not publicly available because they are derived from patient radiographic records and are subject to ethical and privacy restrictions imposed by the Institutional Review Board and applicable data protection regulations.
